# Exploring mutual aid-based community engagement during the COVID-19 pandemic: a case study of community group buying in China

**DOI:** 10.3389/fpubh.2025.1628887

**Published:** 2025-12-10

**Authors:** Weiwei Li, Zhiyuan Yu

**Affiliations:** 1 School of Philosophy and Social Development, Shandong University, Jinan, China; 2 School of Journalism and Communication, Shandong University, Jinan, China

**Keywords:** community engagement, mutual aid, community group buying, COVID-19, WeChat group

## Abstract

**Background:**

Community mutual aid is a form of community engagement involving multiple stakeholders working together to provide both material and emotional support. During the COVID-19 pandemic in China, community group buying (CGB) exhibited distinct features of community mutual aid, reflected in its flexible product supply and spontaneous neighborhood support. However, the mechanisms driving the emergence of such mutual aid practices remain insufficiently explored.

**Methods:**

This study employed a qualitative research design combining ethnographic observation and interpretive case analysis to examine whether, why, and how CGB in China transformed into community mutual aid practices during the lockdown, as well as the outcomes of this transformation. Data were collected primarily through semi-structured interviews (*N* = 12) and participant observation within WeChat groups.

**Results:**

The analysis identified six interrelated dimensions shaping the transformation of CGB into community mutual aid: disaster risk, community networks, participation pathways, psychological motivations, community resilience, and social inequality. Disaster risk acted as an external catalyst that activated social capital, while pre-existing community networks served as internal driving forces—together forming the prerequisites for this transformation. Participation pathways and psychological motivations further facilitated the reproduction of structural and relational social capital, constituting the core process of transformation. This transformation strengthened community resilience by reinforcing trust, reciprocity, and collective efficacy, but also revealed the “dark side” of social capital, as digital divides and unequal access to resources deepened social inequality.

**Conclusion:**

Our findings offer new insights into CGB-based mutual aid in context of China’s crisis. This study not only contributes to the theoretical understanding of mutual aid–based community engagement, but also offers practical insights for mobilizing social capital in future public health emergencies. Further research should investigate the applicability of the CGB model across diverse sociocultural contexts and assess its long-term impact.

## 1 Introduction

Mutual aid has become a prevalent way for people to assist each other in meeting their basic needs through care and solidarity (1). This approach can address the shortage of resources and services in disasters (2). According to the Centers for Disease Control and Prevention, community engagement is the process of working collaboratively with groups of people who are affiliated by geographic proximity, special interests, or similar situations with respect to issues affecting their well-being (3). As a form of community engagement, community-based mutual aid reflects collaborative efforts within neighborhoods. During the COVID-19 pandemic, this practice became particularly vital, taking diverse forms such as mutual care (4), livelihood coping (5), and information sharing (6). As a typical engagement practice in the community, mutual aid can be organized among community residents (7) to meet the requirements of material and mental/physical health (5), alleviate anxiety (8), strengthen neighborhood unity (2), and foster community resilience (9, 10). However, despite the growing prevalence of such practices, a systematic model of community engagement centered on mutual aid remains underdeveloped in academic research.

The pandemic created a global context for observing community engagement, giving rise to diverse response models. Resident-led health protection initiatives relied on spontaneous but fragmented individual coordination (11); government-coordinated response mechanisms allocated resources through top-down administrative systems (12, 13); volunteer networks primarily addressed immediate service gaps (14); digital media-based remote collaboration emphasized information dissemination rather than physical distribution (15); culture-oriented public health communication initiatives highlighted the distinctiveness of local cultures (16); and cross-sectoral collaboration integrated resources from multiple stakeholders (17). Although these models alleviated crisis pressures to varying extents, most depended on formal organizational structures or narrowly defined functions and failed to foster spontaneous, sustained horizontal collaboration among neighbors, which is the defining characteristic of mutual aid discussed above.

In contrast, community group buying (CGB) followed a distinct transformation trajectory during the pandemic. As a rapidly emerging form of e-commerce in China, CGB became widely adopted in urban communities. It merges geographic proximity with social networks, relying on group leaders, typically local residents or small business owners such as vendors or shopkeepers, to organize collective purchases of daily necessities among neighbors (18). These group leaders disseminated product information, collected orders, and coordinated logistics through WeChat groups or mini programs. Once residents placed their orders online, group leaders consolidated them and forwarded bulk orders to retailers or manufacturers, who then delivered the goods to designated pickup points the following day (19).

However, during the COVID-19 lockdowns that caused supply chain disruptions and shortages (20, 21), the operational logic of CGB shifted significantly. Commercial efficiency was no longer the sole objective, as mutual aid became the core driving force. Group leaders went beyond their commercial roles, actively establishing supply channels and sacrificing profits to secure community supplies. Residents voluntarily took on distribution tasks, while neighbors shared real-time information about supply and demand, scarce resources, and emotional support through WeChat groups (22, 23). These practices greatly reduced the commercial nature of group buying, transforming it into a participatory and collaborative system rooted in grassroots communities that emphasized mutual aid (20).

Social capital theory provides a useful framework for understanding the shift from commercial exchange to mutual aid. This theory underscores that social networks, trust, and shared norms constitute essential resources that enable social cooperation and collective action (24, 25). Existing research suggests that social capital forms the foundation upon which mutual aid emerges, while mutual aid, in turn, serves as a vital mechanism for maintaining and reinforcing social capital (26). In essence, mutual aid functions as both a process for activating existing social capital and a context for its continual reproduction (26). Drawing on the mutual aid phenomenon observed in CGB and integrating key propositions of social capital theory, we propose that the mechanism through which CGB evolved into a mutual aid-based form of community engagement during the pandemic lies in the activation and reproduction of internal social capital.

Researchers worldwide have investigated various forms of community mutual aid during the COVID-19 pandemic, including its conceptualization (27), motivations (4, 28), strategies (29–31), values (1, 32), and media applications (33–35), wherein the macro-level implications of community mutual aid have been studied. Nevertheless, a significant gap remains in understanding concrete practices at the micro level, specifically, what forms of mutual aid-based community engagement have emerged and how these practices have been implemented during the pandemic. Although CGB engagement models have been extensively investigated in China (36, 37), few studies have examined the role of social capital in this context. Furthermore, while CGB has been recognized as a form of mutual aid (19), the psychological factors driving these practices require further investigation. This study aimed to address these gaps by analyzing community mutual aid through CGB during the lockdown period. The four research questions (RQs) are as follows:

(1) What fundamental features of community mutual aid were manifested through CGB during the COVID-19 pandemic?
(2) Why did CGB emerge as a form of community mutual aid during the COVID-19 pandemic?
(3) How did CGB transform into a form of community mutual aid during the COVID-19 pandemic?
(4) What are the outcomes of CGB as a form of community mutual aid during the COVID-19 pandemic?

This study makes three key contributions to the understanding of community-based mutual aid in crisis contexts. First, it conceptualizes CGB as an emergent form of community mutual aid that transcends market exchange and state intervention. By revealing how CGB evolved through bottom-up mobilization driven by shared risk, reciprocity, and emotional solidarity (19), the study extends mutual aid theory to digitally mediated, everyday community practices. Second, it advances social capital research by illustrating how structural and relational dimensions of social capital jointly sustain collective action under crisis. Pre-existing neighborhood ties, trust, and reciprocal norms enabled resource coordination and collective resilience, while also exposing the “dark side” of social capital in reproducing digital and generational inequalities. Third, it contributes to the literature on digital platforms and community resilience by demonstrating that digital infrastructures, particularly WeChat, function not only as coordination tools but as social infrastructures that facilitate participation, communication, and emotional connection. However, they also introduce new inequalities, underscoring the ambivalent role of technology in shaping equitable mutual aid. Collectively, these findings enrich theoretical understandings of how social capital and digital technologies interact to generate mutual aid-based community engagement. They also offer practical implications for mobilizing inclusive digital infrastructures and strengthening community governance mechanisms in future crises.

## 2 Literature review

### 2.1 Community mutual aid during the COVID-19 pandemic

The cooperation and reciprocity in mutual aid are crucial for the survival of species, contribute to the evolution of societies, and serve as fundamental organizing principles of human communities (38). Mutual aid refers to the formation of self-organized associations in which individuals work together to address shared health or social issues through reciprocal support (39). These organizations typically arise within community settings and consist of neighborhood members (40). While conventional understandings of mutual aid emphasize interpersonal reciprocity between individuals, recent scholarship highlights a crucial shift toward community-level cooperation (5, 29, 41). In crisis contexts such as the COVID-19 pandemic, mutual aid increasingly manifests as collective community action—where residents jointly mobilize resources and organize self-help activities to address shared risks and disruptions, transforming individual reciprocity into community-based resilience (5).

Community mutual aid can be understood as an organizational form within a community where residents provide assistance through essential provisions, such as food, and emotional support while coordinating to address shared challenges (42). This includes supporting marginalized groups and delivering community care during crises (7), the latter being the focus of this study. Disaster risk is defined as the potential for loss of life, injury, or damage to assets within a system, society, or community over a specific period (43). Community members experienced unprecedented feelings of fear, uncertainty, and confusion amid disaster risks, which in turn motivated mutual aid actions (44). Previous studies have highlighted the importance of community mutual aid in crisis response, including disasters such as Hurricanes Katrina, Sandy, Maria (40), as well as the 2005 London bombing (45). It supports local residents and strengthens community activities at the micro level (9).

During the COVID-19 pandemic, community mutual aid became a central form of community response and a means of strengthening social connections (29). In the United Kingdom, for example, more than 4,000 mutual aid groups were established during the early stages of the pandemic (46). These groups offered various types of support, including the procurement and delivery of essentials, prescription collection, meal provision, pet care, informational sharing, emotional support through helplines, and food bank management (47). By combining information exchange, mutual care, and the mobilization of local resources, these community initiatives collectively addressed residents’ everyday challenges (5). It is noteworthy that early mutual aid was often linked to anarchist perspectives (32); however, during the pandemic, such community-based mutual aid evolved beyond that context and displayed plural and pragmatic orientations—some were entirely grassroots, others partly supported by local governments or embedded within market infrastructures (2). This evolution illustrates that mutual aid has transcended ideological boundaries, becoming a flexible framework for public participation that integrates civic, state, and market logics (48).

Community mutual aid during the pandemic displayed several defining characteristics. First, spontaneity and grassroots participation were key features; when community members faced shared threats, mutual aid groups typically emerged from the bottom up rather than through administrative directives (40). Second, mutual aid groups often maintained flat organizational structures, operating flexibly through neighborhood networks and digital platforms such as WeChat and Facebook (30, 33). Third, mutual aid acted as a resource integrator, mobilizing internal community resources, such as food, medicine, labor, skills, and information, to achieve internal redistribution and relative self-sufficiency (5, 10). Fourth, mutual aid networks provided not only material assistance but also comprehensive care systems through emotional support and information sharing (29, 42, 49). Fifth, reciprocity and collaboration formed its operational logic, emphasizing “solidarity over charity” (50). Finally, emotional motivation was a key sustaining factor. Participants experienced joy through helping others, which reinforced community identity and sustained network operations (28, 29).

Social capital theory provides a crucial theoretical foundation for understanding community-based mutual aid. Coleman conceptualizes social capital as resources embedded within individuals and collectives that influence behavior through particular social structures, thereby generating benefits in specific contexts. Social capital is inherently productive, serving explicit instrumental purposes—individuals can deliberately mobilize it to achieve desired goals (24). Building on Coleman’s insights, Putnam defines social capital as features of social organization—such as networks, norms, and trust—that facilitate coordinated action and cooperation for mutual benefit (25). As a driver of civic engagement, social capital encourages participation in voluntary organizations, which in turn fosters norms of reciprocity and interpersonal trust, ultimately sustaining collective well-being (51).

Banks extends this framework to the domain of mutual aid, arguing that in contexts characterized by individualism and weakened social ties, mutual aid groups maintain participation primarily through their internal social capital (26). Drawing upon Coleman and Putnam’s perspectives, he distinguishes two interrelated dimensions of social capital. The first, relational social capital, is characterized by networks grounded in shared norms and trust that enable individuals to overcome collective action dilemmas such as the “tragedy of the commons” and to pursue shared interests. The second, structural social capital, concerns the configuration and density of social networks through which individuals gain influence and access to resources via exchanges. Together, these two dimensions form the foundation of community mutual aid.

Empirical research further demonstrates that mutual aid practices during the COVID-19 pandemic operated through pre-existing structural networks within communities and relied heavily on relational social capital—particularly trust and reciprocity (30). Simultaneously, the mutual aid process contributes to the reproduction and reinforcement of these forms of social capital: members expand their personal support networks through assistance exchanges (52), while the mechanism of “healing through helping” strengthens trust and reciprocity within the community (26).

### 2.2 Understanding community group buying

CGB is a novel retailing model that combines online e-commerce with offline community retailing, involves many stakeholders (e.g., group leaders, consumers, and retailers) (53) and is supported by CGB platforms (e.g., WeChat, Meituan, and Pinduoduo) (54). Notably, Meituan and Pinduoduo are central to China’s CGB ecosystem, operating through sub-brands such as Meituan Youxuan and Duoduo Maicai. These platforms provide group leaders with standardized product catalogs, logistics support, and integrated payment systems (55). By leveraging technology and capital, they offer advantages in supply chain integration and service standardization, thereby becoming deeply embedded in the everyday consumption practices of local communities (18).

However, compared to these large-scale platforms, WeChat plays a more fundamental and ubiquitous role in the CGB ecosystem (56). On the one hand, most CGB platforms offer mini-program interfaces within WeChat, enabling users to browse products, place orders, and complete payments without the need to install additional apps. On the other hand, in areas with limited or no platform involvement, many CGB activities are organized entirely through WeChat groups, where group leaders manually manage product listings, order collection, and delivery coordination (53).

This self-organized operational form within the WeChat ecosystem reflects a higher degree of grassroots participation, flexibility, and social embeddedness (57). Research shows that CGB in China has effectively leveraged social tools such as WeChat group chats and mini-programs to cultivate strong neighborhood ties, thereby accelerating the growth of the community-based digital economy (58).

**Figure 1:**
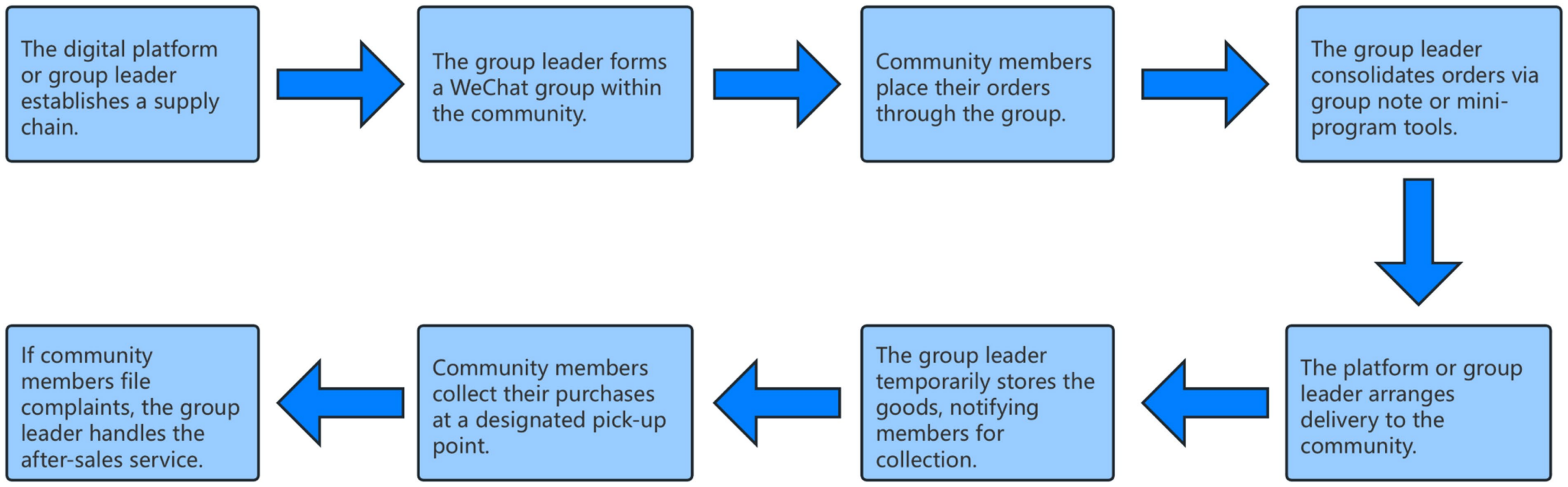
Operational mechanism of CGB.

To further illustrate the operational mechanism of CGB within WeChat-based networks, Figure 1 presents a typical workflow from supply chain establishment to after-sales service management. The process involves multiple actors—including digital platforms, group leaders, and community members—who collaboratively coordinate procurement, distribution, and communication through WeChat groups or mini-program tools (37).

As a promising retail platform model, the sales characteristics of CGB are closely related to consumer psychology (36). Consumers are driven by factors such as perceived value, including convenience and time savings (49); perceived trust in the quality of services offered by the group leader (59); online platforms (36); and good distribution models (60). Importantly, Zhang and Tsai find that collectivist consumer psychology significantly influences purchase decisions in CGB (61). Studies have also scrutinized the impact of digital platforms and highlighted how their features (e.g., convenience, usefulness, and ease of use) influence consumer attitudes and purchase intentions (53, 54, 62). The dual role of group leaders, bridging community ties and commercial transactions, crucially affects perceived trust and consumer behavior (37, 59).

During the COVID-19 pandemic, CGB surged in popularity, particularly in cities such as Wuhan and Shanghai, where it was deployed to address emergency needs and ensure community welfare (63). This process typically involves establishing online platforms, setting community norms, appointing group leaders, and optimizing logistical procedures (19). Stakeholders, including group leaders, residents, estate managers, and volunteers, collaborate to create supply chains, organize food distribution, and enhance community resilience (63). Participation motives are driven by both individual interests and communal benefits, thus fostering community cohesion and increasing local governance engagement (19).

### 2.3 Social media group support in crisis

Social media groups are integral components of platforms such as Facebook, Flickr, and WeChat (64), which form virtual communities where individuals share interests and networks linked with family members, friends, or colleagues (65). These groups typically consist of group owners (in charge of group management) and members (who join through open acceptance, authorization, or invitation) (66).

During crises, social media groups provide three types of social assistance. Instrumental support includes concrete aid and services that help restore communal life in affected areas by mobilizing resources and services within the group (67). Second, informational support enables disaster-affected individuals to share information, ask questions, and solicit advice, thus facilitating knowledge exchange and situational awareness (67). Finally, emotional support allows members to express empathy, affection, confidence, and compassion (68). For example, during the Camp Fire disaster, individuals in a Facebook group shared uplifting quotes and focused on mental health, recovery, personal growth, and appreciation (68). During the COVID-19 pandemic, these social media communities broke through time and space restrictions and enabled individuals to chat online (69). Community managers (e.g., neighborhood committees and grid administrations) have specific responsibilities in each community. They disseminate information related to community-based preventive measures to these groups (70). It is important to note that during this period, the circulation of Mis/Dis/harmful Information (MDHI), as emphasized in the RCRC framework, posed significant challenges. Therefore, community managers not only provided preventive information but also played a role in countering misinformation and maintaining trust within the community (71). Additionally, these groups serve as platforms for residents to seek help, acquire necessities, engage in public affairs, and alleviate loneliness caused by isolation (72). Research has indicated that participation in these groups can enhance residents’ trust and attachment to their community, encouraging greater involvement in community activities (69). In summary, residents’ WeChat groups became essential tools for community outreach during the pandemic, connecting community networks, maintaining operations, and shaping community dynamics (72).

## 3 Methodology

This study employed a qualitative methodology, grounded in an interpretive case study approach, integrating ethnography with semi-structured interviews. The ethnographic approach, including immersive participant observation within CGB WeChat groups during the COVID-19 lockdown, enabled the researcher to gain an in-depth understanding of group dynamics, social interactions, and collective practices (73). Semi-structured interviews were conducted with CGB participants to provide complementary insights into their experiences, perceptions, and affective responses (74). All collected data were systematically organized and coded using NVivo 14 software to facilitate rigorous analysis.

### 3.1 Case selection

As COVID-19 rapidly spread worldwide, many countries adopted lockdown measures in accordance with the WHO guidelines to curb virus transmission (75). In April 2022, the government of Jinan, China, implemented community-based lockdown measures in response to the local COVID-19 outbreak. In this context, two notable cases of CGB emerged in Community W. Community W is a pseudonym used to protect participants’ privacy. It is also the residence of the first author, which facilitated immersive observation and direct interaction with community members, enabling the collection of rich, contextually informed data. To address potential biases associated with insider research, we implemented rigorous qualitative procedures, including systematic data collection, coding, and participant validation, to ensure the credibility and reliability of our findings.

On April 24, 2022, a resident (with the No. L01, Table 1) created a WeChat group called the FeiFei Group for CGB, which included 498 members, all residing in Community W. Five days later, another resident (No. L02) established a similar WeChat group known as the AA Group, which comprised 410 members from the same neighborhood. Both “FeiFei” and “AA” are pseudonyms used to represent these two CGB WeChat groups.

**Table 1:** Participants information.

Name	Gender	Age	Identity	Group buying frequency
L01	Female	35	Group leader	/
L02	Female	44	Group leader	/
M01	Male	32	Group member	18
M02	Female	48	Group member	17
M03	Female	40	Group member	22
M04	Female	22	Group member	9
M05	Male	41	Group member	15
M06	Female	28	Group member	11
M07	Male	52	Group member	6
M08	Male	34	Group member	17
M09	Female	39	Group member	25
M10	Male	40	Group member	8

These two cases were purposefully selected for the study for the following reasons: First, they represent predominant CGB forms emerging in Chinese cities during COVID-19. Model 1, which is characterized as “small and scattered” and initiated by residents (57), is exemplified by the Feifei Group. This model leverages group leaders’ personal networks to establish independent supply chains involving suburban producers and small wholesalers, primarily distributing fresh produce. Despite flexibility and cost advantages, this model faces significant coordination challenges. In contrast, the AA Group represents Model 2, which operates through commercial platforms such as Meituan (76). This model ensures a stable supply of diverse goods, including daily necessities, through centralized operations, but it may limit community decision-making autonomy. These contrasting organizational logics provide a theoretical basis for analyzing the adaptability and limitations of CGB models in crisis contexts.

Second, while large platforms such as Meituan and Pinduoduo constitute major actors in China’s CGB ecosystem, our study focuses on CGB practices led by local group leaders and coordinated through WeChat social networks (19). This focus allows us to investigate the micro-level interaction mechanisms, mobilization strategies, and the reproduction of community engagement logics within the group buying process, rather than the centralized and commercialized operations typical of platform economies. Both selected cases exemplify this form of grassroots, WeChat-based CGB, characterized by spontaneity, flexibility, and close-knit community engagement, which directly aligns with the objectives of our study.

Third, the selection of Feifei Group and AA Group as case studies aligns with the abductive logic adopted in this study. Abductive reasoning emphasizes starting from observed phenomena and inferring the most plausible causes or mechanisms to generate new theoretical insights (77). Field observations in Community W revealed that, despite their independent operational models and organizational structures, these two groups were relatively large, representative, and exhibited a notable similarity in mutual aid patterns. This unexpected phenomenon serves as the starting point for abduction: Why did CGB spontaneously display a consistent logic of mutual aid during the pandemic? Guided by social capital theory, we conducted a comparative analysis of organizational structures, decision-making processes, and member interactions, focusing on the common factors that facilitated mutual aid behaviors. Through a systematic examination of similarities and differences, we abductively inferred the core mechanisms driving mutual aid practices in CGB and constructed a practice-rooted, generalizable theoretical framework.

### 3.2 Procedures

After the formation of the two WeChat groups, we promptly joined both and obtained permission from the group leaders to issue a recruitment announcement. This announcement outlined two criteria for participation: residing in the neighborhood and involvement in at least one CGB activity. Interested individuals were offered a modest compensation as an incentive for participation, but not contingent on providing any specific responses. To minimize the risk of participants joining solely for the compensation, we verified eligibility by confirming prior participation in CGB activities and residence within the community.

As shown in Table 1, 12 participants were voluntarily recruited, including 2 group leaders and 10 members (7 women and 5 men), aged between 22 and 52 years (M = 37.92, SD = 8.35). Additionally, 70% of the participants engaged in 10 or more group purchases. To ensure anonymity, informed consent was obtained from all participants, who were also informed of the research purpose.

From April 24 to November 30, 2022, ethnographic data were collected through immersive participant observation within a CGB WeChat group. Before commencing ethnographic observation, informed consent was obtained from both the group leaders and group members. The group leaders provided verbal consent, and no members expressed objections to the study. All chat records, images, and interaction data were collected solely for academic research, with all personal identifiers removed to ensure participants’ confidentiality. The researcher actively participated in daily interactions, discussions, and group-purchasing activities, documenting digital communication including text, images, videos, voice messages, emojis, and hyperlinks. To enhance reflexivity and minimize researcher bias, reflective field notes were maintained throughout the study, recording personal perceptions and potential influences on data interpretation.

The two CGB groups studied were established during the pandemic; thus, direct ethnographic observation was only possible during this period. After May 15, 2022, when strict community lockdowns in Jinan were largely lifted, the context shifted to the post-pandemic stage. To reconstruct a time-bound comparison across stages, pre-pandemic practices were inferred from participants’ retrospective accounts, supplemented with literature and media reports illustrating general CGB operations before the crisis. Post-pandemic observations and archival data (e.g., WeChat group histories) captured the persistence or decline of mutual aid practices and the reversion to commercial functions. This triangulated approach provided a comprehensive understanding of CGB mechanisms and social dynamics across the pre-pandemic, pandemic, and post-pandemic stages, highlighting their functional evolution.

Therein, we conducted semi-structured interviews with the participants. The interviews lasted between 30 and 60 min, with an average of 48 min. They were conducted both online and offline, in compliance with social distancing guidelines. Online interviews were conducted using WeChat’s voice communication features, whereas offline interviews were primarily conducted during the pickup of CGB merchandise. Following the interview outline, we posed a series of semi-structured questions, as shown in Appendix 1, covering topics such as buyers’ motives, experiences, tools, and perspectives on CGB.

Since all interviews were conducted in Chinese while the manuscript is written in English, we provide an explanation of the translation process. Data analysis was conducted based on the original Chinese transcripts to avoid semantic bias (78). During the writing stage, the first author translated the relevant excerpts into English, and the corresponding author performed back-translation (79). Both authors are bilingual. When encountering expressions with multiple possible translations or culture-specific meanings, we referred to relevant literature, discussed until consensus was reached, and developed a consistent glossary (e.g., translating “团长” as “group leader” and “接龙” as “group note”). To ensure that the translations accurately reflected participants’ intended meanings, we also conducted member checking with selected participants (80).

We adopted an abductive analytical perspective to code and interpret the interview data (77). This approach integrates the systematic, theory-informed procedures of grounded theory—open coding, axial coding, and selective coding—with abductive reasoning as its core logic (81). Abductive reasoning allows iterative movement between empirical data and theoretical frameworks, continuously comparing, refining, and optimizing interpretations to identify the most plausible explanations for observed phenomena (77). This approach is particularly suitable for our study, as it enables the use of social capital theory as a sensitizing concept rather than a predetermined framework, providing heuristic guidance without constraining theoretical development (82). All interview recordings were fully transcribed and imported into NVivo 14 software for a systematic three-stage coding process.

The coding process followed a systematic three-stage procedure. During open coding, we read transcripts line by line, identifying and labeling initial concepts while writing analytical memos to capture emerging insights. For example, the statement, “I think it’s because we are all neighbors, and we trust information that comes from our neighbors (M08 group member),” was coded as “neighborhood relations.” In the subsequent axial coding stage, relationships among concepts were examined and organized hierarchically into sub-categories and main categories. For instance, “neighborhood relationship” was grouped under the sub-category “networks of acquaintance,” which, guided by social capital theory, was further categorized under “community networks.” During this stage, we employed abductive theory matching, iteratively comparing data with theory to explore underlying mechanisms. During the selective coding phase, drawing on the conceptualization of social networks within social capital theory, “community networks” were identified as part of the core category “the causes of community mutual aid,” reflecting their role in facilitating collective assistance. Other main categories were integrated into two additional core dimensions: “the process of community mutual aid” and “the outcomes of community mutual aid.” Together, these core categories formed a theoretical framework explaining the mechanisms of community mutual aid in CGB.

Throughout the analysis, we maintained theoretical openness. For example, the statement, “I was able and felt obligated to help my neighbors during a period of panic when the supermarkets were empty (L01 group leader),” was initially coded as “responsibility” and later refined into the sub-category “sense of rescue.” Although this psychological motivation extends beyond conventional social capital theory, it was incorporated into the broader category “psychological motivations,” alongside reciprocity, reflecting underlying emotional and moral drivers of community mutual aid.

Two researchers conducted independent coding and met regularly to discuss discrepancies, which were resolved through constant reference to the original data (83). All participants were invited to review the research findings. Most confirmed the accuracy of the coding and agreed with our interpretations. For divergent opinions, we revisited the original transcripts and refined coding categories or their relationships where necessary, while retaining these differing perspectives to capture the diversity of experiences and deepen our understanding of mutual aid mechanisms (84).

## 4 Results

This section presents the empirical findings derived from the analysis of CGB during the COVID-19 pandemic. Specifically, it identifies six main categories—disaster risk, community networks, participation pathways, psychological motivations, community resilience, and social inequality—that captured the causes, processes, and outcomes of the transformation of CGB into a form of community mutual aid. Each main category comprises several subcategories and open codes, as detailed in Table 2.

**Table 2:** The main categories, sub-categories, and initial concepts.

Main categories	Sub-categories	Initial concepts	Number of nodes
Disaster risks	Material scarcity	Material shortage	17
		Unstable supply	2
		Fear	9
	Spatial isolation	Lockdown management	11
		Community lockdown	4
		Loneliness	1
Community networks	Networks of acquaintance	Neighborhood relations	9
		Acquaintance society	7
		Social network	13
		Opinion leader	1
		Community norm	11
	Hubs of social support	Material support	13
		Informational support	13
		Emotional support	4
		Trust	13
Participation pathways	Technology mobilization	Technical tools (min-program, group note)	12
		Virtual scenes	4
		Purchase methods	7
	Negotiations of emotions and interests	Nonprofit	4
		Bidirectional feedback	5
		Altruistic tendency	3
Psychological motivations	Sense of rescue	Responsibility	22
		Reciprocity	2
	Sense of community	Membership	10
		Accomplishment	2
Community resilience	Alleviating community challenges	Mitigating material shortages	19
		Providing companionship	6
	Fostering mutual-aid spirit	Community cohesion	7
		Primary-level organization	8
Social inequalities	Material supply uneven	Lack of diversity	1
		High cost	3
	Digital access inequality	Vulnerable groups	2
		Neglected	2

Additionally, these core categories describe how the broader practice of community mutual aid, exemplified by CGB functions. Community mutual aid practices are typically initiated by disaster risks and are based on community networks. These practices rely on public participation strategies and psychological motivations such as reciprocity. While community mutual aid enhances community resilience to disasters, it can also exacerbate social inequalities.

**Figure 2:**
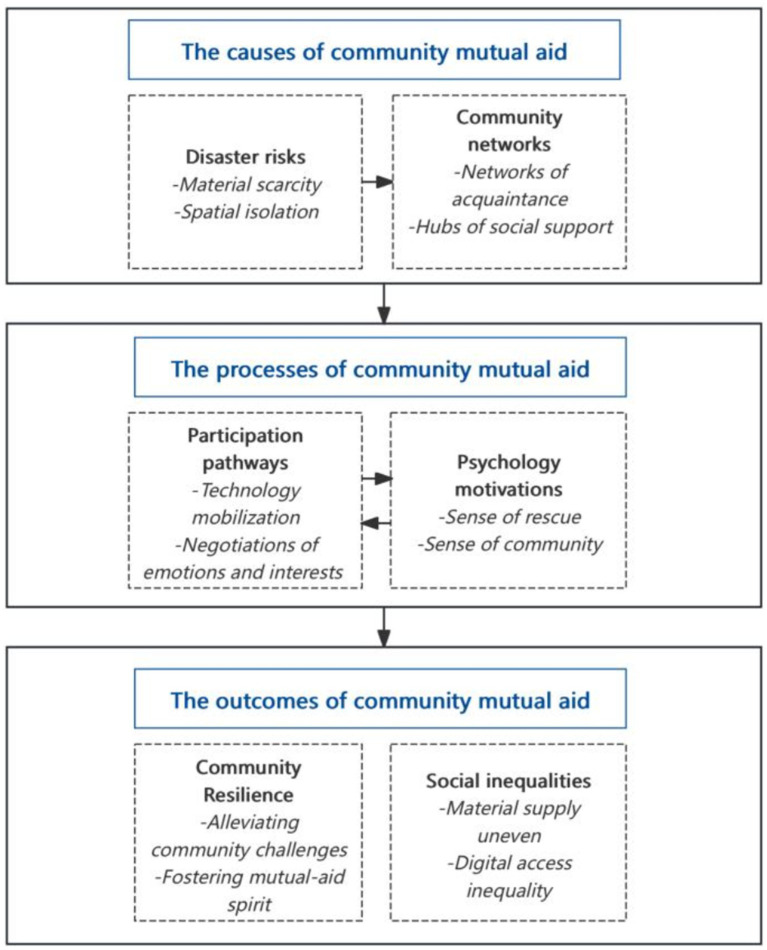
The CGB-based community mutual aid framework.

By synthesizing existing literature and research findings, and following an abductive logic, we developed a theoretical framework of community mutual aid practices, represented by the CGB framework (Figure 2). The developed model illustrates the interconnections between core categories. A recursive relationship exists among the causes, processes, and outcomes of community mutual aid. Specifically, disaster risks strengthen community networks. Participation pathways bolster psychological motivations, which further support participation pathways.

### 4.1 Disaster risks

The COVID-19 pandemic posed a significant risk to individuals’ health and well-being (75). To curb the spread of COVID-19, governments implemented emergency lockdown measures according to the WHO guidelines. Some negative impacts on communities and individuals occurred, such as the temporary disruption of market activities and cessation of express delivery services. Consequently, residents faced multiple disaster risks, such as the scarcity of essential resources and physical isolation.

“The food at the community store was rapidly sold out, and I had not stored any at home.” (M07 group member).

“The community gates were closed, preventing any of us from leaving.” (M06, group member).

Uncertainty is an inherent aspect of disaster risk that cannot be ignored (85). Previous research indicated that uncertainty surrounding the COVID-19 pandemic may lead to persistent and intense fear (86). Additionally, the lockdown intensified feelings of loneliness due to heightened uncertainty (87). Our findings showed that residents experienced significant fear and loneliness during the lockdown period.

“We were afraid, both due to the infection and the prospect of being unable to get food.” (M09 group member).

“Staying at home all day without any interaction with others may be quite lonely.” (M05 group member).

### 4.2 Community networks

Residents form acquaintance networks on WeChat groups based on their existing relationships. These networks also serve as hubs of social support, where residents can offer assistance to one another. CGB is often facilitated through WeChat groups, which can include anywhere from 3 to 500 members (88). Membership in these WeChat groups requires invitations from current members and thus relies on personal connections between new and existing members. This network of acquaintances on WeChat plays a crucial role in facilitating CGB.

“This community buying group on WeChat is composed of all residents of our community.” (M02 group member).

This network is characterized by a lack of clear hierarchical structures and a significant degree of equality among its members. Group leaders, who are often the most influential individuals within the organization, act more as initiators than managers, and do not hold prominent positions within the community network. Group members voluntarily follow rules such as refraining from spreading rumors or mistreating others, rather than being forced into compliance. This structure emphasizes unity and equal status among members, which is consistent with other models of community mutual aid.

“The group leader typically updates the group buying information, and it is rare for them to remove members from the group.” (M01, group member).

In China, the social structure is sometimes described as an “acquaintance society” where interpersonal connections are primarily local with minimal movement among social groups. However, modernization has weakened these acquaintance relationships in urban communities (89). During a shared crisis, the emotions and capacity to gather resources among neighbors within a social network of acquaintances are revived. The Chinese proverb “A close neighbor is preferable to a distant cousin” underscores the reliability of neighborhood support during emergencies.

“We feel like family during these critical situations rather than neighbors.” (M08 group member).

The network structure of social relationships significantly affects the functional content of social support (90). In CGB WeChat groups, the network of acquaintances among residents functions as a hub for social support, thereby facilitating community mutual aid. Four primary forms of social assistance emerged within this network.

The primary and crucial element is instrumental support, in which the group leader provides supplies to the residents, and the residents also provide supplies to each other.

“Within the group, I reached out to those in trouble, and other neighbors were quick to help. Everyone pulled together to overcome difficulties.” (M04, group member).

Second, residents served as information nodes, gathering firsthand information about the pandemic from other networks and sending it to the AA and FeiFei groups. Thus, community networks provide informational support.

“When a confirmed case arose in any of the nearby neighborhoods, everyone instantly informed the group.” (M09 group member).

Third, the community network creates a secure environment for residents to express their emotions, which helps mitigate feelings of isolation and anxiety.

“It was not so scary when I sometimes talked to others in this group.” (M10 group member).

Finally, trust within a community network is crucial beyond typical forms of social support. Trust in group leaders forms the foundation for instrumental, informational, and emotional support, especially in commercial activities such as CGB (49).

“The food acquired by community organizations was both affordable with excellent quality. I trusted the group leader.” (M01 group member).

### 4.3 Participation pathways

Two main aspects of how residents participate in CGB exist: first, they engage in CGB through the utilization of low-cost technologies, such as mini-programs and group notes within WeChat groups, and second, they negotiate their emotions and interests during the group purchasing process.

Mini-programs are crucial technological tools for facilitating CGB. As components of WeChat, mini-programs can be utilized without the need to download or install them, which provides convenient accessibility by allowing users to open them effortlessly through swipes or searches (91).

The AA Group employs a mini-program called the “Vertical Order King.” The mini-program’s homepage displays essential details, such as the leader’s ID (L02), the offered products, their prices, the associated images, and pickup date. Users can easily add products to their shopping cart by clicking the “+” button and complete the checkout process by entering the recipient’s name, phone number, WeChat ID, pick-up point, and other necessary information.

A mini program enhances the shopping experience by offering various features. From the leader’s perspective, this includes product presentations, price labeling, and a user interface for completing purchases. From a consumer’s perspective, it provides a range of products to choose from, a one-click purchase button, and a streamlined checkout screen. These features streamline the purchasing experience and increase the likelihood of purchasing.

“The mini-program operates similarly to a small retail center, with clearly marked products and prices.” (M07 group member).

**Figure 3:**
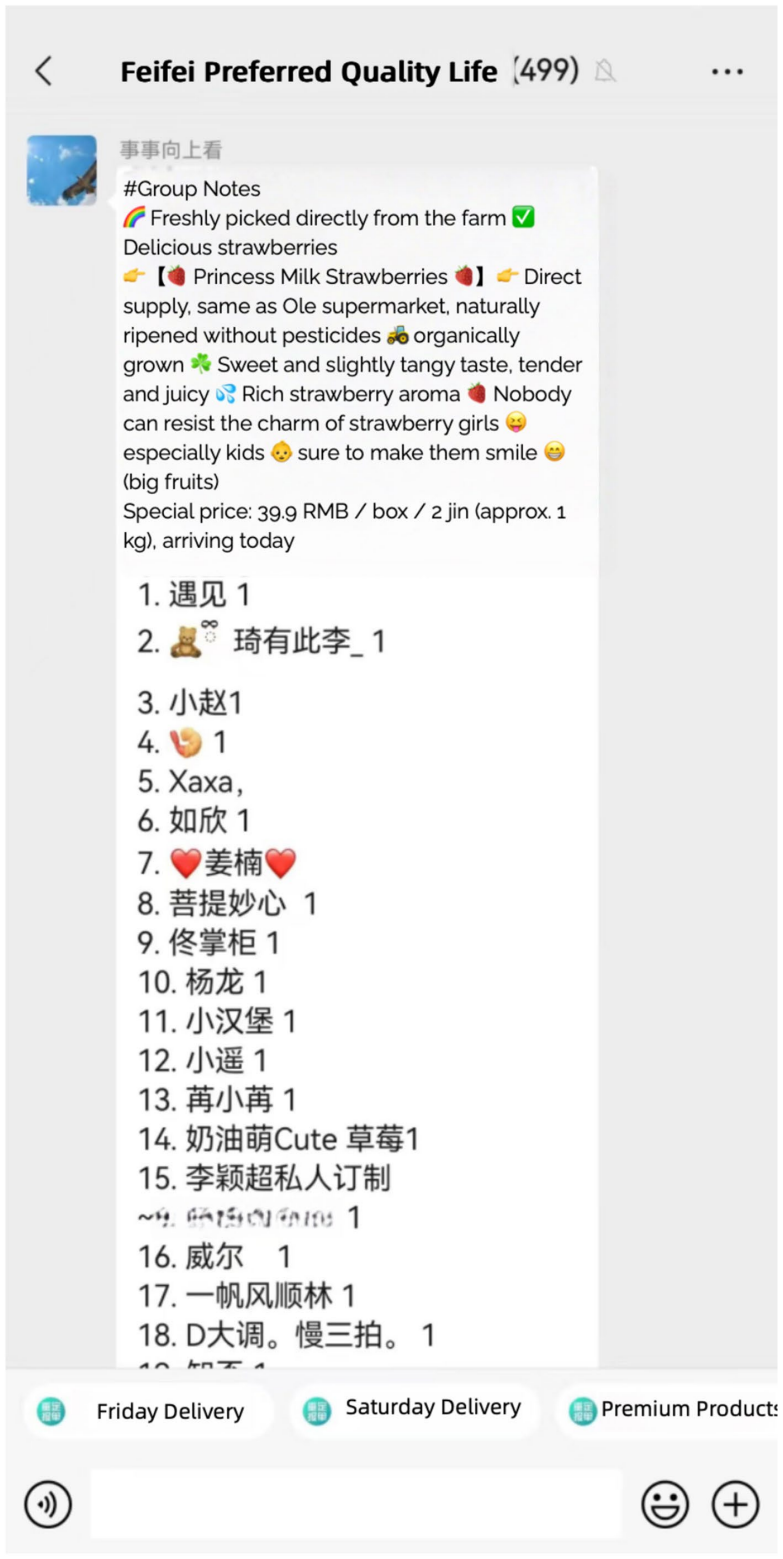
The group note. Chinese characters represent the online nicknames of community residents placing orders, while Arabic numerals indicate the quantity of items requested.

The FeiFei Group uses the “Group Note” feature to monitor statistical data of the group leader and members’ participation in group buying (Figure 3). The group leader disseminates product information by creating notes within the WeChat group. Users can engage by selecting the “#Group Note” button in the group chat, which allows them to track the sales data for various goods visually.

“When I join the WeChat group and click in, I notice that everyone is engaged in the group note. Sometimes, I make purchases without knowing what others are buying, but I still participate.” (M02 group member).

Integrating mini-programs that simulate the shopping experience or group notes that provide a clear view of collective purchasing, coupled with effective technology distribution, establishes a cost-effective mobilization method within the CGB. Moreover, within CGB, group leaders and members negotiate their respective interests. During the COVID-19 lockdown, group leaders often demonstrated altruistic intentions by sacrificing a portion of their earnings to assist others within the community.

L02, a mother of two, initiated a CGB business to leverage its flexibility and manage her time efficiently. Her original intention was to ensure the project sustainability by offering inexpensive prices. However, as the lockdown progressed, she aimed to utilize her resources to help her neighbors overcome challenges regardless of her earnings.

In turn, purchasers often perceive buying products from their neighbors as a means of supporting them. Mutual assistance reflects a community’s collective efforts to manage its interests and emotions.

“By taking part in a CGB led by L02, we can assist L02, a full-time mother, in launching her own business.” (M08 group member).

This negotiation process enables the establishment of a consensus and subtle management of the interests and emotions of buyers and sellers in the CGB. For example, buyers commonly express satisfaction with purchased items through images and written remarks. To incentivize the group members to contribute valuable ideas, as a symbol of gratitude, the group leader distributed digital red packets to the WeChat group.

“Although the quantity of money in the red envelope is not significant, it serves as a token of appreciation.” (M02 group member).

### 4.4 Psychological motivations

In CGB, residents derive psychological motivation from two main factors: a perceived sense of rescue and a strengthened sense of community. Upon receiving notifications about the lockdown, residents of Community W experienced a decrease in the availability of goods in nearby superstores and marketplaces. L01 organized a collective purchase of cucumbers from her suburban garden and sold them at a significantly lower price. When asked about her motivation for initiating CGB, she stated:

“I was able and felt obligated to help my neighbors during a period of panic when the supermarkets were empty.”

Group members who engaged in CGB perceived it as an effective approach to address the scarcity issue when “eggs and vegetables had been depleted and were not accessible.” (M10 group member). Group members understood that the group leaders would profit from these purchases, indicating a reciprocal relationship.

“Goods purchased through CGB are significantly less expensive than those from outside the neighborhood. Group leaders, who are also neighbors, need to make money. So why not purchase from neighbors instead of paying a premium price through other channels?” (M03 group member).

CGB has emerged as a pivotal catalyst in fostering a sense of community among neighbors. The detection of a COVID-19 case in a nearby community would trigger a lot of information (news, screenshots, audio clips, and videos) across CGB WeChat groups. While some residents initially felt alarmed by the sudden influx of information and confirmed cases, others took steps to alleviate the tensions. This period marked a significant shift, in which residents felt a profound connection to their community, emphasizing a sense of inclusion and membership.

“As a member of our community, I am dedicated to guaranteeing its security by sharing information.” (M01 group member).

For critical prevention moments, community officers allow some residents to leave their homes. These individuals subsequently organized transportation teams to facilitate the delivery of collectively purchased goods to residents’ homes. These acts of mutual aid not only fulfilled practical needs, but also instilled a deep sense of accomplishment among participants, further strengthening community bonds.

“It is quite fulfilling to witness everyone saying ‘thank you’ at that time.” (L02 group member).

### 4.5 Community resilience

When CGB evolved into a mutual aid practice, it significantly enhanced the community’s resilience during the lockdown. First, it alleviated community challenges. On the one hand, the material shortages were mitigated within the community, particularly by ensuring the provision of food and other essential needs for residents. Organized efforts to source and distribute goods mean that, even in times of crisis, the basic needs of the community are met, thereby reducing stress and uncertainty. As one participant noted,

“CGB really solved the problem of not having enough food and daily supplies in our neighborhood—it was the most urgent need at the time.” (M04 group Member).

On the other hand, WeChat groups used for organizing these purchases provided a platform for residents to interact, reducing feelings of loneliness and panic. These virtual interactions became a crucial lifeline for emotional support, allowing residents to share experiences, offer help, and build a sense of togetherness despite physical isolation. As reflected in one participant’s words,

“I’m feeling kind of lonely being the only one at home, but the group buying chat is really active. it makes me feel less alone.” (M04 group Member).

Second, the CGB fostered a spirit of mutual aid among residents. In tense and high-pressure environments, residents supported each other by strengthening community solidarity and cohesion. This spirit of mutual support involved not only sharing resources but also building trust and mutual respect among neighbors.

“It greatly improves the convenience of shopping. During times of panic, neighbors support and respect each other through group buying. This brings us closer and more united than before.”(M01 group member).

Initiating CGB on WeChat involved collaboration with neighborhood committees and community grid managers, indicating government involvement in supporting these activities during the pandemic (L02 group leader). Government officials and property managers were involved in ensuring smooth operation of the CGB. Their involvement ensured that logistical challenges were effectively managed, and communication channels between residents and authorities were open. This demonstrated that the community mutual aid initiative received support from both internal and external sources, thereby developing a strong spirit of mutual aid among residents.

### 4.6 Social inequalities

Inequality in the community mutual aid system posed by the CGB also has several negative effects. First, the supply of materials was uneven. Group leaders made efforts to mobilize resources to meet residents’ needs for food and other daily necessities. However, a notable shortage of critical anti-epidemic materials, such as masks and alcohol, occurred temporarily. This scarcity resulted in prohibitively high prices of anti-epidemic materials purchased by community groups.

“CGB has drawbacks such as its inability to procure masks and alcohol, which must still be allocated by the government.” (M01, group member).

Second, inequality existed in terms of digital access. Not all residents of Community W joined WeChat groups for CGB, with some continuing to rely on other retail channels. Our survey found that this group mainly included older adults with limited access to digital tools, along with renters who lacked the social connections needed to be invited into WeChat groups. These vulnerable groups are excluded from the CGB-based mutual aid system.

## 5 Discussion

This study investigates whether, why, and how CGB emerged as a form of mutual aid–based community engagement during the COVID-19 pandemic, and further analyzes the outcomes of this transformation. The analysis identifies six interrelated factors—disaster risk, community networks, participation pathways, psychological motivations, community resilience, and social inequality—that shaped the evolution of CGB. Together, these factors illuminate the operational logic of CGB in crisis contexts and provide a robust empirical foundation for addressing the study’s core research questions.

### 5.1 Mutual aid characteristics of CGB during the COVID-19 pandemic

This study demonstrates that, amid the COVID-19 pandemic, CGB evolved from a pragmatic procurement mechanism into a grassroots model of mutual aid, fully embodying the core features of community mutual aid.

First, the emergence of CGB was neither state-mandated nor market-driven; it represented a form of social self-rescue rooted in residents’ immediate needs and collective survival instincts. Motivated by shared risk perceptions, ordinary community residents spontaneously organized procurement, distribution, and last-mile delivery. This bottom-up mobilization bypassed formal institutions (19), relying instead on informal trust networks and reciprocal norms characteristic of social capital (24, 25). These dynamics illustrate how embedded social capital can be activated to sustain community vitality during crises (92).

Second, CGB adopted a flat, decentralized structure that sharply contrasted with the hierarchical logic of bureaucratic and commercial systems (26). Group leaders acted as coordinators rather than commanders, while members flexibly assumed roles such as information gatherers and volunteers. Digital technologies reduced coordination costs and enhanced agency and equality, illustrating how digital empowerment fosters community resilience amid disruption (35).

Third, CGB WeChat groups functioned as central nodes for resource aggregation and redistribution. Group leaders bridged fragmented market supply and localized demand through collective purchasing and optimized logistics (22). This transformation of individual consumption into collective resource management exemplifies the resource mobilization function of social capital (93), demonstrating how community networks compensate for institutional gaps to maintain social continuity.

Fourth, the dynamics of CGB were grounded in trust and reciprocity. Repeated interactions between organizers and members cultivated enduring obligations and shared norms, which constitute the core mechanisms for the reproduction of social capital. These informal reciprocal norms operated as efficient systems of social regulation (94), promoting cooperation and reducing uncertainty. Over time, they reinforced collective efficacy and facilitated the accumulation of both relational and structural social capital.

Fifth, CGB platforms gradually evolved beyond their initial function of material exchange, becoming multifunctional spaces for social support. Communication channels originally designed for logistical coordination transformed into venues for information sharing and emotional sustenance, consistent with prior findings on digital mutual aid (95). This hybrid online–offline interaction strengthened neighborhood solidarity, alleviated isolation, and fostered a sense of community under conditions of physical distancing (96).

Finally, the enduring vitality of CGB stemmed from its emotional foundations. Amid uncertainty, empathy, shared hardship, and a collective sense of solidarity emerged as intrinsic drivers of participation (97). What began as pragmatic risk mitigation gradually became a ritual of collective identity imbued with emotional resonance, such as sending red envelopes (98). Emotional energy transformed economic transactions into symbolic acts of mutual aid, illustrating how affective ties sustain the reproduction of social capital during crises.

### 5.2 The causes of CGB as a form of community mutual aid during the pandemic

Having established that CGB functioned as a form of community mutual aid during the pandemic, this section examines the underlying conditions that enabled this transformation. Understanding why CGB evolved into mutual aid helps clarify the broader circumstances under which mutual aid–based community engagement emerges.

From a social capital perspective, this transformation was not accidental but driven by the activation of social capital within disaster contexts. Disaster sociology emphasizes that disasters are not merely physical events but structural shocks at the societal level, revealing and amplifying vulnerabilities and inequalities within social systems (7, 8). During the COVID-19 pandemic, for instance, lockdown measures led to shortages of essential goods, exposing weaknesses in food supply chains and intensifying feelings of isolation and anxiety (9). Existing research further suggests that such perceptions of disaster risk can activate social capital, thereby encouraging mutual aid at the community level (10). Disaster risk can therefore be understood as an external catalyst that transformed CGB from an economic exchange network into a mutual aid practice, as members mobilized shared resources under common risk perceptions to establish responsive systems of collective support.

The intrinsic catalyst for this transformation lies in the community’s pre-existing social capital, encompassing established neighborhood networks, shared norms, and trust in local organizers. During the crisis, these elements were reactivated and reintegrated into community interactions, serving as internal driving forces behind the transformation (30). Amid lockdowns and resource shortages, residents utilized digital platforms such as WeChat groups to rebuild social connections and form online mutual aid networks. Although these networks operated through digital media, their core participants were primarily drawn from existing neighborhood ties, reflecting the characteristics of a society of acquaintances. These networks exhibited a horizontal, closed, and stable social structure that embodies structural social capital, wherein residents mobilize and coordinate social resources through established relational networks.

Meanwhile, neighborhood norms were reactivated and gradually institutionalized through ongoing processes of resource coordination and responsibility allocation, giving rise to informal institutions that sustained the mutual aid order. As the Chinese proverb goes, “A good neighbor is better than a distant relative,” highlighting that neighborhood-based mutual aid norms, rooted in acquaintance networks, exhibit strong emotional cohesion and resource mobilization capacity during crises (99). Trust, as another critical form of social capital—particularly institutional trust in community organizers—constituted a key mechanism enabling CGB to maintain operations and fulfill its mutual aid functions during crises. This aligns with the concept of relational social capital (26), which emphasizes social networks grounded in shared norms and mutual trust.

### 5.3 Processes driving the transformation of CGB into community mutual aid

This section explores how CGB transformed into community mutual aid during the pandemic. We compare the evolution of this model before, during, and after the pandemic. As shown in Table 3, it initially functioned as a market-oriented procurement mechanism, shifted to mutual aid practices during the pandemic, and later emerged as a hybrid form combining commercial efficiency with community cohesion. This trajectory reflects functional adaptation rather than organizational restructuring. The shift from a commercial to a mutual aid model was primarily enabled by the reproduction of structural and relational social capital, with digital participation pathways and psychological motivations playing pivotal roles in this process.

**Table 3:** Evolution of CGB before, during, and after the COVID-19 Pandemic.

Comparison dimension	Before the pandemic	During the pandemic	After the pandemic	Key events/mechanism changes
Context/Formation	Driven by daily retail needs; focused on cost efficiency and convenience.	Triggered by emergency life-sustaining needs due to lockdowns and restricted mobility.	Returns to routine consumption, combining convenience with residual mutual aid practices.	Lockdowns and supply shortages triggered emergency CGB and mutual aid; after the pandemic, CGB largely returned to its commercial function, while certain mutual aid practices, such as information support, partially persisted.
Core objective	Enhances cost efficiency and provides neighborhood convenience.	Ensures access to daily necessities through community mutual aid.	Maintains convenience while sustaining some community support.	The focus shifted from purely transactional to securing essential goods; after the pandemic, commercial objectives resumed.
Role of group leader	Disseminates information, collects orders, and assists with fulfillment.	Acts as a central hub and organizer, assuming greater coordination and risk responsibilities to guarantee supply.	Balances commercial and community roles, occasionally coordinating mutual aid or community initiatives.	The role evolved from information distributor to coordinator and mutual aid organizer; post-pandemic, group leaders balance business and community responsibilities.
Community member participation	Place orders and pick up goods.	Place orders, provide material, emotional, and informational support, and some residents spontaneously assist with delivery.	Primarily transactional, with some voluntary mutual aid or neighborhood support persisting.	Participation expanded from purely transactional to active mutual aid; post-pandemic, transactions dominate, but mutual aid habits partially persist.
Transaction tools	WeChat groups (mini-programs, group notes), emphasizing transactional functions.	WeChat groups (mini-programs, group note), emphasizing emergency support functions.	WeChat groups and apps maintain both transactional and occasional support functions.	Tool usage shifted from purely transactional to supporting mutual aid; post-pandemic, both functions coexist, but transactions remain primary.
Revenue model	Group leaders earn commissions and supply chain profits.	Group leaders reduce or forego earnings to ensure the supply of essential goods.	Group leaders resume profit-making, occasionally contributing time or resources for community support.	The revenue model was temporarily altered to prioritize mutual aid over profit; post-pandemic, profit motives resumed.
Psychological motivation	Group leaders seek profit; community members pursue low cost and convenience.	Sense of rescue (responsibility, reciprocity) and sense of community(membership, accomplishment)	A combination of profit motivation and residual sense of community belonging and reciprocity.	Motivation shifted from purely commercial to including mutual aid and belonging; post-pandemic, both motivations coexist.
Outcome/Impact	Provides a new consumption channel for residents.	Mitigates material shortages and strengthens community resilience.	Maintains convenience while fostering sustained community cohesion.	The impact expanded from commercial consumption to include community mutual aid; post-pandemic, a mixed social and commercial outcome remains.

First, at the technological level, digital participation pathways facilitated the reproduction of structural social capital. Previous studies suggest that mutual aid practices increasingly depend on digital platforms to achieve social objectives, as the hybrid digital–physical spaces they generate constitute a vital foundation for forming and reproducing of social capital (100). Consistent with this, digital platforms became central arenas for regenerating social capital within CGB. Through these platforms, individuals were organized, resources mobilized, and social bonds maintained (33–35).

WeChat, for instance, served as the primary medium for CGB. It operated not merely as a technological tool but as a form of social infrastructure embedded in everyday life. As Joyce notes, digital infrastructures—comprising networks, code, applications, and devices—perform structural functions in social mobilization and resource coordination (101). During lockdowns, WeChat’s architecture and functionalities—such as group chats, payment systems, and mini-programs—significantly strengthened community cohesion. Its open application programming interfaces (APIs) further enabled participation from external organizations and users (102), embedding additional forms of social capital, including governmental and commercial resources, into community networks. However, digital platforms are not neutral conduits of mutual aid. As state, market, and public interests intersect within platform spaces, the reproduction of social capital also exposes institutional tensions. Platform algorithms and governance mechanisms can generate power asymmetries, constrain community autonomy, and potentially undermine the equity and sustainability of mutual aid efforts (88). Thus, digital platforms function simultaneously as the infrastructure for reproducing structural social capital and as potential sources of its fragility.

Second, psychological motivations fostered the reproduction of relational social capital. Consistent with prior research (1, 6, 10, 28, 33), residents’ participation in mutual aid during crises stems from a reciprocal mindset—a core element of relational social capital. This sense of reciprocity cultivates empathy and emotional support among members while guiding participants to balance individual and collective interests.

In the context of CGB, participants pursue personal needs—such as securing daily necessities—while simultaneously upholding collective interests by adhering to group norms, engaging in mutual assistance, and participating in volunteer services. This alignment of self-interest with collective well-being encourages residents to internalize community-oriented values, thereby deepening reciprocity and trust among members and mitigating collective action dilemmas such as the tragedy of the commons (26).

Consequently, although community mutual aid networks retain the form of material exchange, their operational logic during the pandemic transcended market rationality. This finding aligns with prior research emphasizing that mutual aid embodies non-commodified social relations (5). Furthermore, the psychological motivations sustaining participation extend beyond reciprocity to include a sense of responsibility, membership, and accomplishment. Viewed through the lens of mutual aid as healing (26), these psychological dimensions enhance both the stability and the emotional resilience of mutual aid networks.

This finding calls for a deeper engagement with Banks’ conceptualization of structural and relational social capital in the context of digitalization and crisis (26). In traditional community settings, these two dimensions of social capital often operated hierarchically: structural capital facilitated coordination, while relational capital sustained commitment (26, 103). However, the results of this study reveal that within the CGB model, the boundary between these dimensions has become increasingly fluid. WeChat-based infrastructures, while primarily serving structural functions by organizing the flow of goods and information, simultaneously cultivate relational ties through everyday interactions. Neighbors, for instance, build mutual trust and reciprocity through digital gestures such as sharing information, embedding emotional exchanges within structural coordination and thereby generating what may be termed hybrid social capital. This hybridization extends Banks’ framework by demonstrating that in digital crisis mobilization, structural and relational social capital are not merely complementary but mutually constitutive. Structural connectivity afforded by digital tools enhances relational trust, while affective reciprocity, in turn, reinforces network efficiency. Thus, the CGB model exemplifies how social capital has evolved into a digital form of community resilience within emerging sociotechnical and crisis contexts (104).

### 5.4 Outcomes of CGB in community mutual aid

The transformation of CGB into community mutual aid produced dual effects: it strengthened community resilience while simultaneously amplifying social inequalities.

Consistent with previous studies (7, 28, 29), our findings indicate that CGB, as a form of community mutual aid, significantly enhanced community resilience—not only by alleviating short-term hardships but also by reinforcing the spirit of solidarity among residents. The social capital cultivated during the crisis facilitated collective mobilization (26), and with support from external factors such as government agencies, this spirit of mutual aid further expanded. Although initially temporary, these effects may prove enduring. Just as community mutual aid during the COVID-19 pandemic relied on preexisting resources, future forms of community mutual aid may have activated the spirit of mutual assistance developed during this period.

However, the study also reveals a “dark side” of social capital: its potential to reproduce social inequality (105). Although CGB embodies the defining features of community mutual aid—operating as a bottom-up, informal support mechanism that complements top-down government aid (6, 7, 28)—it does not benefit all residents equally. While some research suggests that mutual aid can mitigate inequality through the accumulation of social capital (2, 9), our findings indicate that, in certain contexts, these very mechanisms may unintentionally deepen disparities. Such inequalities are particularly pronounced among older adults. Consistent with prior research (106), the digital divide—an enduring form of structural inequality—is especially evident in mutual aid activities that rely heavily on digital platforms such as WeChat groups and mini-programs. As one interviewee noted, many older adults residents were unable to place orders independently and had to depend on government supplies (M02 group members).

This suggests that, while digital platforms have strengthened social cohesion and enhanced the efficiency of mutual aid within certain groups, they have also exacerbated intergenerational disparities in access to information, participation, and resources (107). Moreover, although informal assistance systems like CGB can partially relieve pressure on formal aid mechanisms, they may inadvertently weaken these systems’ responsiveness to vulnerable populations. Formal institutions may assume that all individuals benefit equally from community-based mutual aid, thereby overlooking structural challenges rooted in unequal distributions of social capital (108). These findings highlight the need for future research on community mutual aid to examine inclusivity and redistribution mechanisms within social capital, ensuring that mutual aid practices strengthen, rather than reproduce social inequality.

### 5.5 Implications

This study highlights the substantial potential of resident-led mutual aid initiatives in responding to future public health emergencies. Community mutual aid can effectively mitigate the collective action problem and promote reciprocal benefit, as neighborhood reciprocity plays a vital role in enhancing cohesion and fostering resilience during crises (109). Nevertheless, inequalities in supply distribution and participation barriers—particularly among vulnerable groups such as the older adults remain a pressing concern. To improve the effectiveness of mutual aid and reduce its potential drawbacks, governments and communities are encouraged to institutionalize community mutual aid programs, establish transparent distribution protocols, and provide digital training or offline support to ensure equitable participation. Furthermore, cultivating a sustained culture of mutual aid among residents can contribute to resilient community governance in China (19, 69). Policymakers should therefore strengthen these efforts by providing institutional and technological support for community-based initiatives.

Additionally, this study underscores the essential role of digital platforms in coordinating participants, mobilizing resources, and providing social support during crises. Neighborhood digital platforms, particularly WeChat groups, serve as key infrastructures for sustaining mutual aid networks and community cohesion (110). Community managers can leverage the functional affordances of these platforms to foster engagement and enhance resilience, while authorities should focus on empowering digital tools and ensuring equitable access—especially in marginalized communities—to maximize the benefits of digital inclusion for grassroots governance.

### 5.6 Study limitations

This study had some limitations. First, during the pandemic, a variety of community-based mutual aid approaches emerged across different regions. Although the CGB model captures shared themes, it is insufficient to comprehensively represent the diverse practices of mutual aid and solidarity observed in communities worldwide. Second, we employed an interpretive case study approach to examine the community mutual aid process based on the CGB model. This methodological choice resulted in a relatively small sample size and primarily descriptive data, limiting the generalizability of the findings. Third, this study acknowledges a key limitation in its underrepresentation of older adults and the lack of systematic data concerning this demographic. Therefore, future research should focus on two specific areas: (1) evaluating the applicability of the CGB model across different sociocultural contexts, (2) assessing the post-pandemic impact of CGB and its contribution to strengthening community resilience and (3) expanding the sample by including focused in-depth interviews and customized questionnaires specifically designed for older participants.

## 6 Conclusion

This study investigates how community group buying (CGB) evolved from a commercial procurement mechanism to a form of community engagement grounded in mutual aid during the COVID-19 pandemic. Its core characteristics include spontaneous grassroots organization, flat and decentralized structures, resource integration and redistribution, trust- and reciprocity-based interactions, functional expansion, and emotional motivation. This transformation was driven by the activation of social capital under crisis conditions: externally triggered by disaster risks and supply shortages, and internally sustained by pre-existing structural social capital (e.g., neighborhood networks providing coordination frameworks) and relational social capital (e.g., shared norms and trust in organizers). Digital platforms functioned as social infrastructures facilitating the reproduction of structural social capital. Meanwhile, residents’ psychological motivations—including awareness of reciprocity, sense of membership, accomplishment, and responsibility—served to reinforce relational social capital. This process produced dual outcomes: enhancing community resilience by alleviating hardships, strengthening solidarity, and laying the foundation for future collective action, while also intensifying intergenerational digital divides and social inequalities.

## Data Availability

The original contributions presented in the study are included in the article/Supplementary material, further inquiries can be directed to the corresponding author.

## References

[1] Littman DM Boyett M Bender K Dunbar AZ Santarella M Becker-Hafnor T . Values and beliefs underlying mutual aid: an exploration of collective care during the COVID-19 pandemic. J Soc Soc Work Res. (2022) 13:89–115. doi: 10.1086/716884

[2] Rendall J Curtin M Roy MJ Teasdale S. Relationships between community-led mutual aid groups and the state during the COVID-19 pandemic: complementary, supplementary, or adversarial? Public Manag Rev. (2024) 26:313–33. doi: 10.1080/14719037.2022.2084769, 38818046 PMC11138323

[3] Centers for Disease Control and Prevention. Community Engagement Playbook 32. (2021). Available online at: https://www.atsdr.cdc.gov/community-engagement-playbook/php/introduction/glossary.html [Accessed February 25, 2023].

[4] Mao G Drury J Fernandes-Jesus M Ntontis E. How participation in COVID-19 mutual aid groups affects subjective well-being and how political identity moderates these effects. Anal Soc Issues Public Policy. (2021) 21:1082–112. doi: 10.1111/asap.12275, 34899075 PMC8652987

[5] Carstensen N Mudhar M Munksgaard FS. 'Let communities do their work': the role of mutual aid and self-help groups in the COVID-19 pandemic response. Disasters. (2021) 45 Suppl 1:S146–73. doi: 10.1111/disa.12515, 34562282 PMC8653332

[6] Ntontis E Fernandes-Jesus M Mao G Dines T Kane J Karakaya J . Tracking the nature and trajectory of social support in Facebook mutual aid groups during the COVID-19 pandemic. Int J Disaster Risk Reduct. (2022) 76:103043. doi: 10.1016/j.ijdrr.2022.103043, 35601394 PMC9106594

[7] Chevée A. Mutual aid in North London during the COVID-19 pandemic. Soc Mov Stud. (2022) 21:413–9. doi: 10.1080/14742837.2021.1890574, 41307611

[8] O'Dwyer E Beascoechea-Seguí N Souza LGS. The amplifying effect of perceived group politicization: effects of group perceptions and identification on anxiety and coping self-efficacy among members of UK COVID-19 mutual aid groups. J Community Appl Soc Psychol. (2022) 32:423–37. doi: 10.1002/casp.2582, 34898965 PMC8653376

[9] Mould O Cole J Badger A Brown P. Solidarity, not charity: learning the lessons of the COVID-19 pandemic to reconceptualise the radicality of mutual aid. Trans Inst Br Geogr. (2022) 47:866–79. doi: 10.1111/tran.12553, 35937505 PMC9347405

[10] Lofton S Kersten M Simonovich SD Martin A. Mutual aid organisations and their role in reducing food insecurity in Chicago's urban communities during COVID-19. Public Health Nutr. (2022) 25:119–22. doi: 10.1017/s1368980021003736, 34462038 PMC8458839

[11] de Oliveira AR. The Brazilian slums hiring their own doctors to fight COVID-19. BMJ. (2020) 369:m1597. doi: 10.1136/bmj.m1597 32321703

[12] Jiang S Zhang D Irwin DD. Semiformal organizations and control during the COVID-19 crisis in China. Asian J Criminol. (2021) 16:75–90. doi: 10.1007/s11417-020-09334-z, 33144893 PMC7595876

[13] Al Siyabi H Al Mukhaini S Kanaan M Al Hatmi S Al Anqoudi Z Al Kalbani A . Community engagement approaches for effective national COVID-19 pandemic preparedness and response: an experience from Oman. Front Public Health. (2021) 8:616763. doi: 10.3389/fpubh.2020.616763, 33575243 PMC7870984

[14] Wickramanayake J. Meet 10 young people leading the COVID-19 response in their communities (2020). Available online at: https://www.un.org/africarenewal/web-features/coronavirus/meet-10-youngpeople-leading-covid-19-response-their-communities [Accessed July 17, 2025].

[15] Hua J Shaw R. Coronavirus (COVID-19) "infodemic" and emerging issues through a data lens: the case of China. Int J Environ Res Public Health. (2020) 17:2309. doi: 10.3390/ijerph17072309, 32235433 PMC7177854

[16] Osborn C. How Brazil's COVID-19 response has fallen to community leaders (2020). Available online at: https://www.thenewhumanitarian.org/news/2020/05/27/Brazil-coronavirus-response-community-leaders [Accessed July 17, 2025].

[17] Gilmore B Ndejjo R Tchetchia A De Claro V Mago E Lopes C . Community engagement for COVID-19 prevention and control: a rapid evidence synthesis. BMJ Glob Health. (2020) 5:e003188. doi: 10.1136/bmjgh-2020-003188, 33051285 PMC7554411

[18] Lin Z Li G Mehmood MS Nie Q Zheng Z. Spatial analysis and optimization of self-pickup points of a new retail model in the post-epidemic era: the case of community-group-buying in Xi'an City. Comput Urban Sci. (2023) 3:13. doi: 10.1007/s43762-023-00089-8, 36970600 PMC10025067

[19] Yang R Qi Y. Neighbourhood governance, citizen initiatives and media application: investigating community group buying during Shanghai's COVID lockdown. Int J Disaster Risk Reduct. (2023) 93:103793. doi: 10.1016/j.ijdrr.2023.103793

[20] Fei S Ni J Santini G. Local food systems and COVID-19: an insight from China. Resour Conserv Recycl. (2020) 162:105022. doi: 10.1016/j.resconrec.2020.105022, 32834481 PMC7318924

[21] Benker B. Stockpiling as resilience: defending and contextualising extra food procurement during lockdown. Appetite. (2021) 156:104981. doi: 10.1016/j.appet.2020.104981, 33038478 PMC7541051

[22] Sigley G Powell W Cao S. Community group buying, vulnerable communities and COVID-19 in China In: Georgeou N Hawksley C, editors. State responses to COVID-19: A global snapshot on 1 June 2020. Penrith, NSW, Australia: Western Sydney University (2020). 50–1. doi: 10.26183/5ed5a2079cabd

[23] Terbeck F He SJ Cai R. Neighborly help and neighborhood-based social capital during the Covid-19 pandemic in major Chinese cities. J Housing Built Environ. (2023) 40:555–75. doi: 10.1007/s10901-023-10076-4

[24] Coleman JS. Social capital in the creation of human capital. Am J Sociol. (1988) 94:S95–S120. doi: 10.1086/228943

[25] Putnam RD. Social capital and public affairs. Bull Am Acad Arts Sci. (1994) 47:5–19. doi: 10.2307/3824796

[26] Banks E. The social capital of self-help mutual aid groups. Soc Policy. (1997) 28:30–9.

[27] Littman DM Morris K Hostetter CR Boyett M Bender K Holloway B . How was mutual aid being conceptualized during its proliferation in the early months of the COVID-19 pandemic? A critical phenomenological analysis. J Community Pract. (2023) 31:193–214. doi: 10.1080/10705422.2023.2210136

[28] Cocking C Vestergren S Ntontis E Luzynska K. 'All together now': facilitators and barriers to engagement in mutual aid during the first UK COVID-19 lockdown. PLoS One. (2023) 18:e0283080. doi: 10.1371/journal.pone.0283080, 37043513 PMC10096193

[29] Fernandes-Jesus M Mao G Ntontis E Cocking C McTague M Schwarz A . More than a COVID-19 response: sustaining mutual aid groups during and beyond the pandemic. Front Psychol. (2021) 12:716202. doi: 10.3389/fpsyg.2021.716202, 34744875 PMC8563598

[30] Travlou P. Kropotkin-19: a mutual aid response to COVID-19 in Athens. Des Cult. (2020) 13:65–78. doi: 10.1080/17547075.2020.1864119

[31] Drury J Fernandes-Jesus M Mao G Ntontis E Perach R Miranda D. How can covid mutual aid groups be sustained over time? The UK experience In: O'Dwyer E Souza LGS, editors. Psychosocial perspectives on community responses to Covid-19. London, UK: Routledge (2022). 79–90.

[32] Jun N Lance M. Anarchist responses to a pandemic: the COVID-19 crisis as a case study in mutual aid. Kennedy Inst Ethics J. (2020) 30:361–78. doi: 10.1353/ken.2020.0019

[33] Bender K Littman DM Dunbar AZ Boyett M Milligan T Santarella M . Emergent media scan of digital mutual aid organizing during the COVID-19 pandemic. J Community Pract. (2021) 29:280–98. doi: 10.1080/10705422.2021.1980477

[34] Knearem T Jo J Tsai C-H Carroll JM. Making space for support: an exploratory analysis of pandemic-response mutual aid platforms. In: Proceedings of the 10th international conference on Communities & Technologies – Wicked problems in the age of tech; 2021 Jun 20–25; Seattle, WA, USA. New York: ACM (2021). p. 38–43

[35] Wilson KR Roskill OM Mahr J. Mutual aid using digital technology: a case study of virtual community organizing during the COVID-19 pandemic. J Community Pract. (2022) 30:255–78. doi: 10.1080/10705422.2022.2102101

[36] Song Y Gui L Wang H Yang Y. Determinants of continuous usage intention in community group buying platform in China: based on the information system success model and the expanded technology acceptance model. Behav Sci. (2023) 13:941. doi: 10.3390/bs13110941, 37998687 PMC10669444

[37] Ying H Ji H Shi X Wang X. A trust model for consumer conversion in community-based group buying: the dual roles of group leaders. Mod Supply Chain Res Appl. (2022) 4:122–40. doi: 10.1108/mscra-01-2022-0004

[38] Kropotkin PA. Mutual aid: A factor of evolution. London: W. Heinemann (1904). 31 p.

[39] Seebohm P Chaudhary S Boyce M Elkan R Avis M Munn-Giddings C. The contribution of self-help/mutual aid groups to mental well-being. Health Soc Care Community. (2013) 21:391–401. doi: 10.1111/hsc.12021, 23445336

[40] Spade D. Solidarity not charity: mutual aid for mobilization and survival. Soc Text. (2020) 38:131–51. doi: 10.1215/01642472-7971139

[41] Ashford RD Brown AM Dorney G McConnell N Kunzelman J McDaniel J . Reducing harm and promoting recovery through community-based mutual aid: characterizing those who engage in a hybrid peer recovery community organization. Addict Behav. (2019) 98:106037. doi: 10.1016/j.addbeh.2019.106037, 31330467 PMC6708724

[42] Soden R Owen E. Dilemmas in mutual aid: lessons for crisis informatics from an emergent community response to the pandemic. Proc ACM Human-Computer Interaction. (2021) 5:1–19. doi: 10.1145/3479862

[43] UNDRR. Disaster risk. Available online at: https://www.undrr.org/terminology/disaster-risk [Accessed January 28, 2023].

[44] Afifi WA Felix ED Afifi TD. The impact of uncertainty and communal coping on mental health following natural disasters. Anxiety Stress Coping. (2012) 25:329–47. doi: 10.1080/10615806.2011.603048, 21801075

[45] Drury J Cocking C Reicher S. The nature of collective resilience: survivor reactions to the 2005 London bombings. Int J Mass Emerg Disasters. (2009) 27:66–95. doi: 10.1177/028072700902700104

[46] Wakefield JRH Bowe M Kellezi B. Who helps and why? A longitudinal exploration of volunteer role identity, between-group closeness, and community identification as predictors of coordinated helping during the COVID-19 pandemic. Br J Soc Psychol. (2022) 61:907–23. doi: 10.1111/bjso.12523, 35122285 PMC9111824

[47] Booth R. Community aid groups set up across UK amid coronavirus crisis (2020). Available online at: https://www.theguardian.com/society/2020/mar/16/community-aid-groups-set-up-across-uk-amid-coronavirus-crisis [Accessed March 16, 2023].

[48] Solnit Rebecca. The way we get through this is together': the rise of mutual aid under coronavirus (2020). Available online at: https://www.theguardian.com/world/2020/may/14/mutual-aid-coronavirus-pandemic-rebecca-solnit [Accessed October 16, 2025]

[49] Bender K Saavedra K Milligan T Littman DM Becker-Hafnor T Dunbar AZ . How mutual aid proliferation developed solidarity and sense of collective responsibility in the early months of COVID-19. Am J Community Psychol. (2023) 73:431–45. doi: 10.1002/ajcp.12721, 37975206

[50] Knearem T Jo J Alliyu O Carroll JM. Solidarity not charity! Empowering local communities for disaster relief during COVID-19 through grassroots support. Comput Supported Coop Work. (2024) 33:559–604. doi: 10.1007/s10606-023-09484-5

[51] Helliwell JF Putnam RD. The social context of well-being. Philos Trans R Soc Lond Ser B Biol Sci. (2004) 359:1435–46. doi: 10.1098/rstb.2004.1522, 15347534 PMC1693420

[52] Höltmann G Hutter S Specht J. How social capital matters for receiving social support: on the complementary role of civil society in the COVID-19 pandemic. Eur Soc. (2023) 25:804–28. doi: 10.1080/14616696.2023.2176528

[53] Tian X Jiang H Zhao X. Product assortment and online sales in community group-buying channel: evidence from a convenience store chain. J Retail Consum Serv. (2024) 79:103838. doi: 10.1016/j.jretconser.2024.103838

[54] Zhu Q Zuo RX Liu S Zhang F. Online dynamic group-buying community analysis based on high frequency time series simulation. Electron Commer Res. (2020) 20:81–118. doi: 10.1007/s10660-019-09380-5

[55] Hongsuchon T Li J. Accessing the influence of consumer participation on purchase intention toward community group buying platform. Front Psychol. (2022) 13:887959. doi: 10.3389/fpsyg.2022.887959, 35837624 PMC9275674

[56] Zhou Y Dong C. Nourishing social solidarity in exchanging gifts: a study on social exchange in Shanghai communities during COVID-19 lockdown. Humanit Soc Sci Commun. (2023) 10:627. doi: 10.1057/s41599-023-02152-5

[57] Stevanovic N Bolaji DA. Fostering resilience: the role of WeChat in grassroots community group buying amidst the COVID-19 lockdown in China. J Creat Commun. (2025):09732586251337569. doi: 10.1177/09732586251337569

[58] Huang X Wu Y. Unveiling trust mechanisms in WeChat Tuangou: a case study of a community-based Tuangou group in China. Open J Soc Sci. (2024) 12:1–13. doi: 10.4236/jss.2024.1212001

[59] Wu J Chen Y Pan H Xu A. Influence of multi-role interactions in community group-buying on consumers' lock-in purchasing intention from a fixed leader based on role theory and trust transfer theory. Front Psychol. (2022) 13:903221. doi: 10.3389/fpsyg.2022.903221, 35783755 PMC9240223

[60] Zhang Z Gu CY. Effects of consumer social interaction on trust in online group-buying contexts: an empirical study in China. J Electron Commer Res. (2015) 16:1–21.

[61] Zhang JJ Tsai W-HS. United we shop! Chinese consumers' online group buying. J Int Consum Mark. (2015) 27:54–68. doi: 10.1080/08961530.2014.967902

[62] Qi Y Shi J Liu Y Tang Z. Research on factors influencing consumers' willingness to use community group buying platform. J Distrib Sci. (2024) 22:1–10. doi: 10.15722/jds.22.05.202405.1

[63] Li B Qian J Xu J Li Y. Collaborative governance in emergencies: community food supply in COVID-19 in Wuhan. China Urban Gov. (2022) 2:188–96. doi: 10.1016/j.ugj.2022.03.002

[64] Kietzmann JH Silvestre BS McCarthy IP Pitt LF. Unpacking the social media phenomenon: towards a research agenda. J Public Aff. (2012) 12:109–19. doi: 10.1002/pa.1412

[65] Wang X Tang L Gao H Liu H. Discovering overlapping groups in social media. In: Proceedings of the 2010 IEEE International Conference on Data Mining; 2011 Jan 20; Sydney, NSW, Australia. Los Alamitos: IEEE Computer Society (2011). p. 569

[66] Albris K. The switchboard mechanism: how social media connected citizens during the 2013 floods in Dresden. J Contingencies Crisis Manage. (2018) 26:350–7. doi: 10.1111/1468-5973.12201

[67] Li J Stephens KK Zhu Y Murthy D. Using social media to call for help in hurricane Harvey: bonding emotion, culture, and community relationships. Int J Disaster Risk Reduct. (2019) 38:101212. doi: 10.1016/j.ijdrr.2019.101212

[68] Brown AR. (re)constructing community after disaster: survivors' use of Facebook groups 1 year after the camp fire. Sociol Inq. (2022) 92:1196–216. doi: 10.1111/soin.12483

[69] You Z Wang M He Z. Residents' WeChat group use and pro-community behavior in the COVID-19 crisis: a distal mediating role of community trust and community attachment. Risk Manag Healthc Policy. (2023) 16:833–49. doi: 10.2147/rmhp.S407534, 37193293 PMC10182810

[70] Pan SL Cui M Qian J. Information resource orchestration during the COVID-19 pandemic: a study of community lockdowns in China. Int J Inf Manag. (2020) 54:102143. doi: 10.1016/j.ijinfomgt.2020.102143, 32394997 PMC7211621

[71] Tjilos M Tamlyn AL Ragan EJ Assoumou SA Barnett KG Martin P . “Community members have more impact on their neighbors than celebrities”: leveraging community partnerships to build COVID-19 vaccine confidence. BMC Public Health. (2023) 23:350. doi: 10.1186/s12889-023-15217-2 36797724 PMC9933023

[72] Chen Y Chen Y Yu S Yu S. Utilizing social media for community risk communication in megacities: analysing the impact of WeChat group information interaction and perception on communication satisfaction during the COVID-19 pandemic in Shanghai. BMC Public Health. (2024) 24:1889. doi: 10.1186/s12889-024-19276-1, 39010017 PMC11247861

[73] Robinson L Schulz J. New avenues for sociological inquiry: evolving forms of ethnographic practice. Sociology. (2009) 43:685–98. doi: 10.1177/0038038509105415

[74] Kallio H Pietilä AM Johnson M Kangasniemi M. Systematic methodological review: developing a framework for a qualitative semi-structured interview guide. J Adv Nurs. (2016) 72:2954–65. doi: 10.1111/jan.13031, 27221824

[75] Peleg K Bodas M Hertelendy AJ Kirsch TD. The COVID-19 pandemic challenge to the all-hazards approach for disaster planning. Int J Disaster Risk Reduct. (2021) 55:102103. doi: 10.1016/j.ijdrr.2021.102103

[76] Zhang M Hassan H Migin MW. Exploring the consumers' purchase intention on online community group buying platform during pandemic. Sustainability. (2023) 15:2433. doi: 10.3390/su15032433

[77] Timmermans S Tavory I. Theory construction in qualitative research: from grounded theory to abductive analysis. Sociol Theory. (2012) 30:167–86. doi: 10.1177/0735275112457914

[78] van Nes F Abma T Jonsson H Deeg D. Language differences in qualitative research: is meaning lost in translation? Eur J Ageing. (2010) 7:313–6. doi: 10.1007/s10433-010-0168-y, 21212820 PMC2995873

[79] Brislin RW. Back-translation for cross-cultural research. J Cross-Cult Psychol. (1970) 1:185–216. doi: 10.1177/135910457000100301

[80] Birt L Scott S Cavers D Campbell C Walter F. Member checking: a tool to enhance trustworthiness or merely a nod to validation? Qual Health Res. (2016) 26:1802–11. doi: 10.1177/1049732316654870, 27340178

[81] Rahmani F Leifels K. Abductive grounded theory: a worked example of a study in construction management. Constr Manag Econ. (2018) 36:565–83. doi: 10.1080/01446193.2018.1449954

[82] Co JCT Loyola DB Martinez EKL Poticano DEP Mabalay AA Daradar DBD . An abductive exploration of sustainability implementation intent among family business successors: extending the theory of planned behavior. Sustainable Futures. (2024) 8:100396. doi: 10.1016/j.sftr.2024.100396

[83] McLellan-Lemal K MacQueen E. Team-based codebook development: structure, process, and agreement In: MacQueen KM Namey EE, editors. Handbook for team-based qualitative research. Lanham, MD: Altamira Press (2008). 119–36.

[84] Naeem M Ozuem W Howell K Ranfagni S. A step-by-step process of thematic analysis to develop a conceptual model in qualitative research. Int J Qual Methods. (2023) 22:16094069231205789. doi: 10.1177/16094069231205789

[85] PateCornell ME. Uncertainties in risk analysis: six levels of treatment. Reliab Eng Syst Safe. (1996) 54:95–111. doi: 10.1016/s0951-8320(96)00067-1

[86] Mertens G Gerritsen L Duijndam S Salemink E Engelhard IM. Fear of the coronavirus (COVID-19): predictors in an online study conducted in march 2020. J Anxiety Disord. (2020) 74:102258. doi: 10.1016/j.janxdis.2020.102258, 32569905 PMC7286280

[87] Shah SGS Nogueras D van Woerden HC Kiparoglou V. The COVID-19 pandemic: a pandemic of lockdown loneliness and the role of digital technology. J Med Internet Res. (2020) 22:e22287. doi: 10.2196/22287, 33108313 PMC7647474

[88] Tu F. Wechat and civil society in China. Commun Public. (2016) 1:343–50. doi: 10.1177/2057047316667518

[89] Tian X Guo Y. An online acquaintance community: the emergence of Chinese virtual civility. Symbol Interact. (2021) 44:771–97. doi: 10.1002/symb.537

[90] House JS Umberson D Landis KR. Structures and processes of social support. Annu Rev Sociol. (1988) 14:293–318. doi: 10.1146/annurev.so.14.080188.001453

[91] Tian T Zhao N. The impact of social media marketing on the dissemination of mini program in social network with different community structure. J Inf Sci. (2023) 51:1081–95. doi: 10.1177/01655515231161523

[92] Aldrich DP Meyer MA. Social capital and community resilience. Am Behav Sci. (2015) 59:254–69. doi: 10.1177/0002764214550299

[93] Hwang H Lee YJ. Community social capital, racial diversity, and philanthropic resource mobilization in the time of a pandemic. City Community. (2023) 22:22–47. doi: 10.1177/1535684122111918 37038511 PMC10076987

[94] Ostrom E. Social capital: a fad or a fundamental concept. Soc Capital A Multifact Perspect. (2000) 172:195–8.

[95] Hultquist C Tubbeh RM. Digital sociotechnical systems of mutual aid: how communities connected, adapted, and innovated during the COVID-19 pandemic in new York City. Citizen Sci Theory Pract. (2022) 7:1–13. doi: 10.5334/cstp.454

[96] Benton E Power A. Community responses to the coronavirus pandemic: how mutual aid can help. LSE Public Policy Review. (2021) 1:1–9. doi: 10.31389/lseppr.2134308354

[97] Slavich GM Roos LG Zaki J. Social belonging, compassion, and kindness: key ingredients for fostering resilience, recovery, and growth from the COVID-19 pandemic. Anxiety Stress Coping. (2022) 35:1–8. doi: 10.1080/10615806.2021.1960676 34369221 PMC8792144

[98] Collins R. "Interaction ritual chains and collective effervescence." In: Scheve C. von Salmela M., editors. Collective emotions. Oxford: Oxford University Press (2014). 299–311.

[99] Chen X Zou Y Gao H. Role of neighborhood social support in stress coping and psychological wellbeing during the COVID-19 pandemic: evidence from Hubei, China. Health Place. (2021) 69:102532. doi: 10.1016/j.healthplace.2021.102532, 33752161 PMC7972939

[100] Qadri R. What's in a network? Infrastructures of mutual aid for digital platform workers during COVID-19. Proc ACM Human-Computer Interaction. (2021) 5:1–20. doi: 10.1145/3479563

[101] Joyce M. Digital activism decoded: The new mechanics of change. New York: IDEA (2010). 14 p.

[102] Li S Xu A Pan X Chen Y Wang Y. Food provision during the outbreak — the WeChat Mini program for material supply platform. In: Proceedings of the 2023 16th International Symposium on Computational Intelligence and Design (ISCID); 2023 Dec; Hangzhou, China. Los Alamitos: IEEE (2023)

[103] Moran P. Structural vs. relational embeddedness: social capital and managerial performance. Strateg Manag J. (2005) 26:1129–51. doi: 10.1002/smj.486

[104] Poortinga W. Community resilience and health: the role of bonding, bridging, and linking aspects of social capital. Health Place. (2012) 18:286–95. doi: 10.1016/j.healthplace.2011.09.017, 22037322

[105] Baycan T Öner Ö. The dark side of social capital: a contextual perspective. Ann Reg Sci. (2023) 70:779–98. doi: 10.1007/s00168-022-01112-2

[106] Martins Van Jaarsveld G. The effects of COVID-19 among the elderly population: a case for closing the digital divide. Front Psych. (2020) 11:577427. doi: 10.3389/fpsyt.2020.577427, 33304283 PMC7693633

[107] Beaunoyer E Dupéré S Guitton MJ. COVID-19 and digital inequalities: reciprocal impacts and mitigation strategies. Comput Human Behav. (2020) 111:106424. doi: 10.1016/j.chb.2020.106424, 32398890 PMC7213963

[108] Lin N. Inequality in social capital. Contemp Sociol. (2000) 29:785–95. doi: 10.2307/2654086

[109] Mollinger I. Community in times of COVID: building resilience in a self-help neighborhood in Medellin. Child Geogr. (2024):1–15. doi: 10.1080/14733285.2024.2352363

[110] Robaeyst B Baccarne B De Meulenaere J Mechant P. Online neighborhood networks: the relationship between online communication practices and neighborhood dynamics. Media Commun. (2022) 10:108–18. doi: 10.17645/mac.v10i2.5129

