# Supplementary material for: Exploring mutual aid-based community engagement during the COVID-19 pandemic: a case study of community group buying in China
Source: Front Public Health. 2025 Dec 10;13:1628887. doi: 10.3389/fpubh.2025.1628887 (PMC12727898; doi:10.3389/fpubh.2025.1628887)
Supplement: Supplementary file 1 [file Table_1.docx]

Supplementary Material

**Appendix 1 Interview Outline**

Thank you for agreeing to do an interview with me today. Your experience is invaluable to us. We are interested in learning about your experiences with community group buying. There are no right or wrong answers, so please feel free to answer as openly and honestly as you can. If you prefer not to answer a particular question, you can request to move on to the next question.

Your responses will be used for academic research and your personal information will be kept confidential. Would you like to continue with the interview?

For the group buying leaders:

(1) What was your initial reason for organizing a community group buying?

(2) What motivates you to continue?

(3) Where do you source your supplies?

(4) What steps are involved in running community group buying?

(5) What challenges did you face during the community lockdown, and how did you deal with them?

(6) Did you receive assistance from sources outside the community during the lockdown?

(7) Did you need approval from the neighborhood council to carry out this work?

(8) Why did you choose to use group notes or mini programs to manage orders or information?

(9) What were your thoughts or feelings about seeing your neighbors' feedback on the products?

(10) In what ways do you believe community group buying has influenced your neighborhood?

For the group buying members:

(1) How do static management policies affect your life?

(2) What motivated you to participate in a community group buying for the first time?

(3) In what ways do you believe community group buying has affected your life?

(4) What differences, if any, do you notice between community group buying and shopping at regular supermarkets?

(5) How did you join the WeChat group for community group buying? What rules or guidelines are required for participating in this group?

(6) Are you comfortable using mini programs or group notes for placing orders?

(7) Do you provide feedback on the products you received from WeChat groups? If yes, what motivates you to do so?

(8) Do you engage in casual conversations within the group? Do you believe that this group serves purposes beyond group buying?

(9) In what ways do you think community group buying affects your neighborhood?

(10) Do you plan to continue participating in community group buying after the pandemic ends?
